# The Patient-Physician Relationship in the Internet Age: Future Prospects and the Research Agenda

**DOI:** 10.2196/jmir.3.2.e15

**Published:** 2001-04-09

**Authors:** Ben S Gerber, Arnold R Eiser

**Affiliations:** ^1^Section of General Internal MedicineCollege of MedicineUniversity of IllinoisChicagoUSA

**Keywords:** Physician-Patient Relations, Internet, Decision-Making, Patient Education, Medical Informatics

## Abstract

In the "Internet Age," physicians and patients have unique technological resources available to improve the patient-physician relationship. How they both utilize online medical information will influence the course of their relationship and possibly influence health outcomes. The decision-making process may improve if efforts are made to share the burden of responsibility for knowledge. Further benefits may arise from physicians who assist patients in the information-gathering process. However, further research is necessary to understand these differences in the patient-physician relationship along with their corresponding effects on patient and physician satisfaction as well as clinical outcomes.

## Introduction

Increasingly, individuals around the world are turning to the Internet for health-related knowledge [1]. In the United States, more than 52 million adults have searched the World Wide Web for health and medical information [2]. An increasing number of health-related Web sites are now becoming available for up-to-date answers to medical questions. In response to this information-seeking activity, physicians have expressed concern regarding access to misinformation and patients' interpretation of available online content [3-9]. Many doctors believe that only qualified medical professionals may adequately assess and interpret external sources of information. Defensive attitudes may arise from the Internet having a "leveling effect" on access to information and, subsequently, on the patient-physician relationship [10,11]. This situation contrasts with physicians' sole possession of medical knowledge, as was the case for most of the 20th century. Today, there is greater acceptance of more informed and educated patients. Healthcare providers can take advantage of this unique opportunity to create, support, reference, and promote awareness of quality electronic sources of medical information. Still, practitioners may differ according to the extent they embrace this technological revolution and make it part of the patient-physician relationship.

In this commentary, we explore the effects of information obtained through the World Wide Web on the patient-physician relationship. The impact of the Web affects decision-making processes and offers new possibilities for physician-to-patient recommendations. However, there remains much uncertainty about what effect the Web has on public health outcomes [12]. Similarly, uncertainty surrounds individual reactions to using Internet information for making medical decisions. With these points in mind, we propose a research agenda for further investigation of online information and its effects on the patient-physician relationship.

## Patient Personality/Information Types in Medical Decision-Making

One of the newest sources of knowledge for patients comes from visiting health-related Web sites. The greatest impact on medical decision-making may come from this increase in knowledge prior to the clinical encounter. Until recently, in the clinical visit the physician had the sole responsibility for medical knowledge, whereas the patient was only accountable for his or her own preferences. Now, by more easily obtaining medical information prior to seeing their doctors, patients potentially have a different position in the decision-making process; possessing both preferences and knowledge prior to any physician contact. Another probable advantage comes from having the opportunity to reflect on and reconsider preferences prior to discussions with health professionals. People are likely to redefine their desires and intentions over time because they frequently are uncertain [13].

Paradoxically, a patient's interest in knowledge may not always accompany an interest in the medical decision-making process. According to studies of patient-physician relationships, although patients typically express a high degree of interest in learning about their illnesses and treatment, their preference for actual participation in treatment decision-making is highly variable [14,15]. Patients may investigate information about their medical conditions without interest in taking responsibility for making decisions about treatment. Given this variability, two kinds of patient-physician encounters may result, based on differential interest in medical decision-making. For each situation, additional knowledge obtained from the Internet offers potential benefit yet may influence decision-making and outcomes in different ways. However, in both of these scenarios the advantages realized assume accuracy in the health information obtained.

### Physician and the Informed Decision-Maker

In one scenario, a patient may be motivated to become involved in the decision-making process and have access to additional sources of information about a particular illness as well as the treatments available (the *informed decision-maker*). Such a patient could be at an additional advantage by having accessed related information via the Internet prior to meeting with a physician. Instead of utilizing scheduled time to provide the patient with basic knowledge, the physician may devote extra time to refining what the patient has learned and offering greater depth on treatment options (assuming the information obtained is accurate). Theoretically, more time could be spent on discussions necessary to arrive at a clinical decision. However, physicians must be prepared to address alternative possibilities that the patient has learned about from external sources. Instead of saving time, this scenario may require extra discussion when untested approaches need to be debunked (as in the case of some complementary and holistic medicine practices). The concept of efficient use of clinical time is of greater importance when the restrictive pressures of managed care and business economics enter the equation. Still, it is yet uncertain whether efficiency improves or declines when patient-acquired Internet information is brought into the decision-making process. This subject warrants further investigation.

The "deliberative" or "participatory" decision-making model is recommended as the preferred model of treatment decision-making in the clinical encounter [14,16,17]. One necessary requirement for this decision-making process is that both parties take steps to participate in the process of treatment decision-making. In this model, the patient takes a newly found responsibility for disclosing preferences, obtaining information, and weighing treatment alternatives. Someone who is willing to accept such responsibility will be at an advantage through consulting the Internet for information. The patient brings to the table technical knowledge in addition to that offered by the physician. This is more likely the case if the information was obtained through a qualified Web site certified by an evaluating organization for accuracy. Eventually, informed consent may become more a reality than a theoretical concept.

The physician's role in shared decision-making has several requirements. Physicians must ensure that the information a patient wishes to use in making a decision is founded in fact and not misconception or falsehood. In addition, proposed treatment options must be weighed with assistance from physicians. To accomplish these tasks, physicians must be prepared to address alternative therapies that may not have been suggested if the patient had not learned about them from external sources.

Health providers must avoid frustration about having their role as the sole source of information challenged, or possibly risk losing patients. In one survey of different specialty and general medicine practices, one third of the patients who felt their relationship with their physicians was low in participatory decision-making changed providers within a year [16]. In addition, because higher volume practices were rated as "less participatory," efficiency becomes an important factor to consider. Thus, physicians must be open to those highly motivated patients who are active participants in their healthcare.

Shared decision-making includes the ideal that both parties need to agree on a treatment option, even if both do not agree that it is the best possible treatment to implement [14]. Certain types of physicians are probably more likely to subscribe to this model than others. Some doctors may not be willing to relinquish the authoritative role. Research suggests that physicians vary widely in the extent to which they feel comfortable in facilitating patient participation in decision-making [16]. In one survey of 1276 Norwegian physicians, 3 out of 4 doctors had experiences with patients bringing Internet information to the consultation setting [18]. Although most found this experience unobtrusive, some believed it had a negative effect on the patient-physician relationship, and others found it to be a positive challenge.

### Physician and the Knowledge-Acquirer

Physicians need to be aware that patients who are interested in obtaining additional knowledge may not be motivated to participate in actual decision-making. This circumstance may reflect less assertive personality traits on the part of some patients. Consequently, the patient-physician relationship may more likely resemble the "physician-as-agent" model [13-14]. In this case, the patient (the *knowledge-acquirer*) provides some personal values to the physician. By possessing the medical knowledge and learning about the patient's values and beliefs, the physician may then be the formulator of the final decision. Though the patient may not actively pursue outside sources of information prior to the clinical visit, there still may be interest in learning more about the medical condition or treatment decided on by the physician. This case was found to be particularly true after relatively long patient-physician encounters [19].

This type of patient may benefit from obtaining information on the Web after the clinic visit. This supplemental information may allow the individual to feel more comfortable or satisfied with a treatment decision, even though there is no involvement in the actual decision-making process. For example, when behavioral interventions are addressed, prior interactions with a physician may have a "priming effect" - improving the behavioral response to reading materials encountered subsequently [20]. This outcome may be a potentially important benefit not realized by physicians who mistakenly feel that "no interest in making decisions" translates into "no interest in medical knowledge." Physicians who recommend Web sites may further benefit patients who acquire medical knowledge via the Internet.

## Physicians Recommending Web Sites to Patients: The Internet Prescription

A physician-recommended Web site could be thought of as an *Internet prescription*. For example, a young woman presents to her physician's office with an interest in starting an exercise regimen, but she is concerned about developing athletic injuries. The Internet-savvy physician "prescribes" a specific Web page on stretching exercises [21]. At home, the patient initially views the recommended information, including images, animation, or video [22]. Subsequently, she also searches the Internet for alternative information and ends up reading about the dangers of traditional stretching exercises [23]. The physician may not have intended her to read this information; though it may be of interest to the open-minded patient. Although healthcare providers may suggest to patients that they acquire information from specific sources, patients will likely obtain a "second opinion" on the Internet. In this case, the potential benefit of the Internet prescription may arise from a patient viewing suggested information first and giving it preference because his or her physician provided it.

Furthermore, patients who find additional sources of information on the Internet have the option of obtaining another opinion through their physicians. In this case, the woman in our example could provide her physician with the Web address (or printed information) that addresses the dangers of traditional exercises. This step may promote discussion between her and her physician about its interpretation. Whereas it is difficult to teach "evidence-based medicine" to the layperson, it is more feasible to discuss articles with patients using related concepts that physicians have learned.

There is great concern about the accuracy and validity of medical information found on the Internet [3-5]. For the physician prescribing Web sites, there is the persistent challenge of ensuring quality in online content. Both physician and patient must become aware of what information is available, the source of information, and the intended audience [24]. Online information that differs significantly from that prescribed by the physician may result in unanticipated consequences. The additional strength and reinforcement of referenced consumer information requires the physician to carefully review what patients will read and to recognize that such information may be periodically updated. In the instance of a major medical illness, some sites may soothe an individual's anxiety whereas others may raise false hopes [25]. The physician's traditional reluctance to offer more information than is necessary may be well intended. However, with the Internet, patients may opt to pursue stories and anecdotal literature evoking strong emotions (for an example, see ConquerCancer.com [26]).

To combat online misinformation, healthcare providers must positively influence patient selection of online materials. The presentation of awards on medical Web pages may not have a significant impact on patients' assessment of credibility [27]. However, approximately 3 out of 4 Internet users seeking health information feel that a doctor recommendation would make them more likely to trust a health Web site [28]. Unfortunately, less than 5% say they currently use doctor recommendations to find the sites visited on the Internet. Physicians need to take an active role in this regard. For example, physicians can link their own Web sites to various known Web sites that provide quality content. This idea appears to be increasing in popularity as physician practice Web sites continue to grow in number. In one corporate survey of over 700 physicians, the percentage of pediatrician practices with Web sites increased from 24% in August 1999 to 46% in October 2000 [29]. As an alternative, medical journals and professional health organizations may represent even more valuable sources, for they offer assessment and dissemination of the best evidence for clinical problems.

Referenced Web sites may be explicitly recommended to patients during clinical encounters or by electronic mail. It then becomes important for physicians to know where high caliber information is located in cyberspace rather than merely know what the specific information is itself [24]. Given how difficult it is for health professionals to keep track of the ever-changing Web, it becomes equally important to know about quality repositories of medical links. The "healthfinder" Web site selects links to health information from sources that include government agencies, nonprofit and professional organizations serving the public interest, universities and other educational institutions, libraries, and so on [30].. This site was developed by the US Department of Health and Human Services to provide up-to-date resources beyond what physicians have time to prepare on their own. The National Library of Medicine has created MEDLINE *plus*, which allows the provider or consumer to search quality Web sites for health information [31]. Physicians may feel more comfortable recommending information from MEDLINE *plus* rather than a "dot-com" source of medical information, which often endorses products or companies.

Despite the existence of quality repositories of health information, there is still significant resistance to online physician activity. In a survey of 1084 physicians by the American Medical Association, only 11% of respondents felt the Internet was useful in providing patient education [32]. This aversion may be related to factors including start-up time, computer/network finances, time spent verifying the accuracy of information on Web sites, and liability issues. Many have a "fight or flight" response to these technical communicative innovations, creating a challenge in implementation [33].

## The Research Agenda

Though there have been previous studies analyzing the patient-physician relationship, research must be directed toward evaluating the impact of electronically obtained knowledge on this relationship. Further analysis of the current models for the patient-physician relationship may reveal that new, emerging trends are taking place. Efficiency, patient satisfaction, and clinical encounter time may vary when Internet-acquired information is considered in decision-making. Variability in patient types and in physician personalities compounds the dynamics of decision-making analysis. Additional focus must be placed on studies that include the impact of electronically obtained knowledge on the patient-physician relationship.

Another issue that should be addressed is the extent of responsibility that a patient is willing to accept. In one pilot study, individuals have been given access to their medical records and have been provided with online communication with their physicians (derived from Web-based methods of sharing clinical content) [34]. Patient interest, as well as physician acceptance, has been evaluated. In another pilot project, patients are being provided with consumer health information in waiting and exam rooms [35]. The resulting patient-physician communication and level of satisfaction will be measured.

When patients assume a greater role in acquiring medical knowledge, there must be a corresponding change in the physician's role as treatment decision-maker. Additional dynamics are likely to result from different physician behaviors, including embracing, avoiding, or disregarding Internet-derived information. To better define this variable, surveys and observational studies are needed that will elicit physician attitudes toward Internet health information and their corresponding patient-physician relationships. In addition, research is needed to evaluate the barriers to physician implementation of information technology. In Canada, researchers have administered a new survey instrument to stratify primary care physicians into different levels of information technology usage [36]. This approach may allow for specifically tailored strategies to be used in implementation.

Although many individuals have the potential to gain medical knowledge easily through on-line information, others do not. Few studies have examined the benefit of computers in patient education within economically depressed urban areas [37]. There is also little evidence that describes how individuals lacking the latest technology (including high-speed Internet Service Providers) cannot access resource-intensive Web sites, including those requiring audio or video streaming. The long-term effects and potential benefits of computer technology for vulnerable populations have yet to be determined. Although there is a considerable amount of data that demonstrate limited access, there still is overwhelming interest in computer education by all segments of the public. Additional research is necessary to define how patients of different cultural or socio-economic backgrounds utilize computers and the Internet for information, and how this has an impact on their relationship with healthcare providers.

Most patients using a home computer have access to medical information on the Internet. This circumstance will likely reflect a select, educated patient population with income levels that support the equipment. In an inner-city medical center in Los Angeles, California, 18% of surveyed minority patients with low levels of income and educational background had Internet access - considerably less than the corresponding national estimate of 37% to 45% [38]. Yet there was significant interest expressed in on-line health information. If minority patient populations are to become active participants in the Internet age, it is necessary to continue to devote greater resources to improving easy access of electronic information. There is a definite need for interventions that empower ethnic minority patients and help them become informed and active healthcare consumers [39].

Patients with poor literacy skills are less likely to take advantage of the Internet in order to acquire additional medical knowledge, whether they have access or not. Unfortunately, because these individuals are more likely to have worse health, their needs for health education are greater, especially for those with chronic illnesses [40]. This issue affects their relationship with physicians; studies have shown that patients' acceptance of diagnoses and treatment plans depends on education [41]. Hence, additional efforts are required to assist persons with lower literacy skills. With adaptive technologies supplying touch-screen input and audio output, kiosks can be made available for patients motivated to learn, independent of their literacy or education level [42,43]. Physician offices with health information kiosks may be an alternative method for browsing health-related information, being temporally linked to clinician interactions. However, additional issues, such as cost, complexity of use, and potential for misinformation, then arise [44]. Still, additional research is necessary to determine the possible benefits and effects on the patient-physician relationship.

In sum, the research agenda on on-line information and the patient-physician relationship includes: (1) an assessment of Internet medical information usage by patients on patient outcomes, satisfaction, and willingness to share decision-making responsibility; (2) determination of changes in physician efficiency, satisfaction, and willingness to share decision-making responsibility; and (3) studies of methods to increase access to computer-based information for patients with low computer and print literacy, which assess process and outcomes measures.

## Conclusions

The Internet Age is altering the patient-physician relationship. If physicians actively assist patients in the information-gathering process, an improved relationship may result. Through the understanding of evolving professional roles, the decision-making process between physicians and patients may improve with efforts to share the burden of responsibility for knowledge. This change could usher in a new era of the patient-physician relationship, with a potential gain for all collaborative parties. However, there is no assurance that implementation will occur smoothly or in a desirable fashion. Thus, there is a compelling need for prospective research in this area. Methods of bridging the Digital Divide are also important considerations for future research, for this disparity in technology use still exists today [45]. It is essential that large segments of the population not be left behind as strides are made in information technology and healthcare decision-making.
